# Metabolic variables of obese dogs with insulin resistance supplemented with yeast beta-glucan

**DOI:** 10.1186/s12917-021-03106-2

**Published:** 2022-01-03

**Authors:** Chayanne Silva Ferreira, Thiago Henrique Annibale Vendramini, Andressa Rodrigues Amaral, Mariana Fragoso Rentas, Mariane Ceschin Ernandes, Flavio Lopes da Silva, Patricia Massae Oba, Fernando de Oliveira Roberti Filho, Marcio Antonio Brunetto

**Affiliations:** 1 Rio Verde University, Fazenda Fontes do Saber, PO Box 104, Rio Verde, Goiás, 75901-970 Brazil; 2 Department of Animal Nutrition and Production, School of Veterinary Medicine and Animal Science, University of São Paulo, 87, Prof. Orlando Marques de Paiva Ave, São Paulo, São Paulo 05508270 Brazil; 3 Grandfood Industria e Comercio LTDA, Luiz Augusto de Oliveira Hwy, km 204, Dourado, São Paulo, 13590-000 Brazil; 4 Biorigin, Lençóis Paulista, São Paulo, Brazil

**Keywords:** beta-glucan, canine, cholesterol, triglycerides, weight loss

## Abstract

**Background:**

Obesity is one of the most common nutritional disorders in dogs and cats and is related to the development metabolic comorbidities. Weight loss is the recommended treatment, but success is difficult due to the poor satiety control. Yeast beta-glucans are known as biological modifiers because of their innumerable functions reported in studies with mice and humans, but only one study with dogs was found. This study aimed to evaluate the effects of a diet supplemented with 0.1% beta-glucan on glucose, lipid homeostasis, inflammatory cytokines and satiety parameters in obese dogs. Fourteen dogs composed three experimental groups: Obese group (OG) with seven dogs with body condition score (BCS) 8 or 9; Lean group (LG) included seven non-obese dogs with a BCS of 5; and Supplemented Obese group (SOG) was the OG dogs after 90 days of consumption of the experimental diet.

**Results:**

Compared to OG, SOG had lower plasma basal glycemic values (*p *= 0.05) and reduced serum cholesterol and triglyceride levels. TNF-α was lower in SOG than in OG (*p *= 0.05), and GLP-1 was increased in SOG compared to OG and LG (*p *= 0.02).

**Conclusion:**

These results are novel and important for recognizing the possibility of using beta-glucan in obesity prevention and treatment.

## Background

Excess body weight (obesity or overweight) is the most common nutritional disorder in dogs and cats [1, 2]. It is estimated that 40 to 60% of the world's canine population is overweight or obese [3, 4], and this percentage has increased progressively over the years [5]. Several clinical chronic conditions are frequently attributed to the condition of being overweight, especially locomotor changes, endocrinopathies, an increased risk for developing neoplasms, and a decreased life expectancy [6, 7].

Since adipocytes are metabolically active and responsible for producing inflammatory cytokines, resistin and leptin [8, 9], being overweight is frequently associated with insulin resistance [10, 11] and metabolic alterations involved in the control of satiety [12, 13]. Recovering an ideal body condition score can reestablish plasma insulin concentrations to values close to the physiological state [14, 15] and reduce inflammatory cytokine production [14], indicating the importance of treating and reducing canine obesity. Successful obesity treatment is defined as the loss of body weight and its effective maintenance afterwards [15, 16]. Thus, inducing a negative energy balance through caloric restriction and increasing the energy expenditure is necessary [17]. According to Weber et al. [15], caloric restriction is considered the major obstacle during weight loss period due to the manifestation of hunger, which consequently leads the animal to seek and beg for food and may often compromise the owner's compliance. Therefore, developing strategies that benefit satiety control are of great interest for managing these animals.

The composition of the diet used in the treatment of obesity, especially regarding to protein and fiber content, contributes significantly for the control of hunger. Beta-glucans are polysaccharides composed of glucose monomers that are linked by β-glycosidic bonds. These polysaccharides are the major structural components of the cell wall of yeasts, fungi and some bacteria. Cereals, such as barley and oats, also contain beta-glucans as part of the cell wall and endosperm [18, 19]. Due to its complex mechanism of action in the body, several effects have already been associated with beta-glucan supplementation in humans, pigs, dogs, rats and fish such as modified immune responses [20, 21], reduced inflammatory responses [22–24], altered glucose [25, 26] and lipids [26, 27] metabolism.

In this context, this work aimed to evaluate the effects of 0.1% beta-glucan dietary supplementation on different glycemic, insulinemic, serum triglyceride, cholesterol, inflammatory cytokines and satiety markers in obese dogs.

### Results

**Fig. 1:**
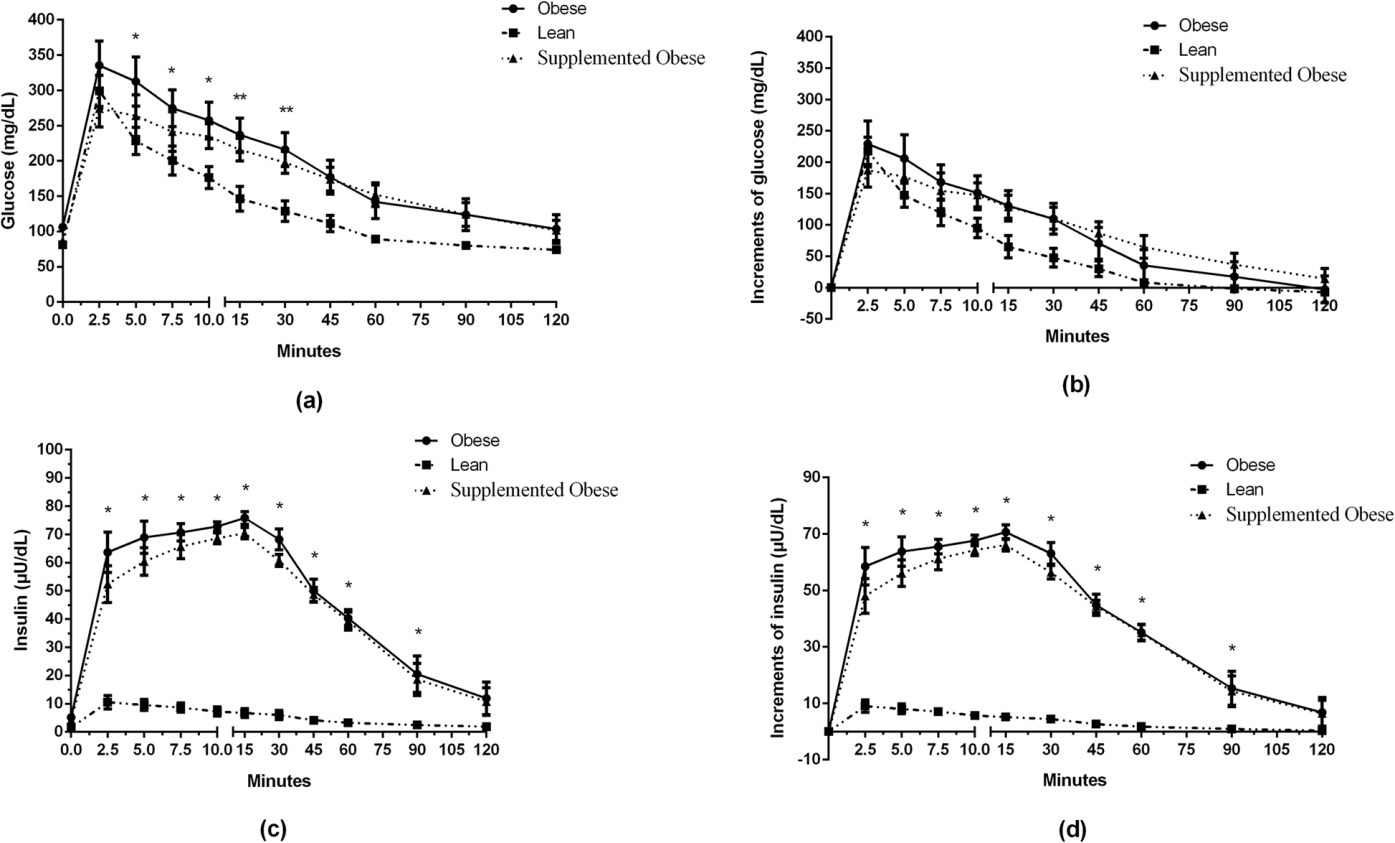
Effect of beta glucan intake on glycemic and insulinemic response. Glycemic curves (**a**), glucose increments (**b**), insulin curves (**c**), and (**d**) insulin increments of the experimental groups

None of the dogs had variations in body weight during the experimental period. In the three experimental groups, glycemic peak was observed at the first collection period (T2.5 min) after glucose infusion (Fig. 1a). At 5, 7.5 and 10 minutes, blood glucose values were lower in Lean group (LG) than in Obese group (OG) but were not different from those in Supplemented Obese group (SOG). At 15 and 30 minutes, the glycemia values in LG were different from those of OG and SOG. After 45 minutes, blood glucose levels had returned to baseline only in LG but not in OG or SOG, even after 120 minutes of testing. In the remaining time, blood glucose values were not significantly different.

Analysis of glucose blood concentration (Fig. 1b) revealed no differences among the groups (*p *> 0.05), but significant changes occurred between time points within the same group (*p *< 0.05), which is physiologically expected and consistent with the normal pattern of glucose absorption.

Insulin values (Fig. 1c) did not differ among the groups at baseline or at 120 minutes. At the remaining time points, insulin values were lower in LG than in OG and SOG (*p *< 0.05). As for the insulin increment (Fig. 1d), the results were similar to those for serum insulin.

**Fig. 2:**
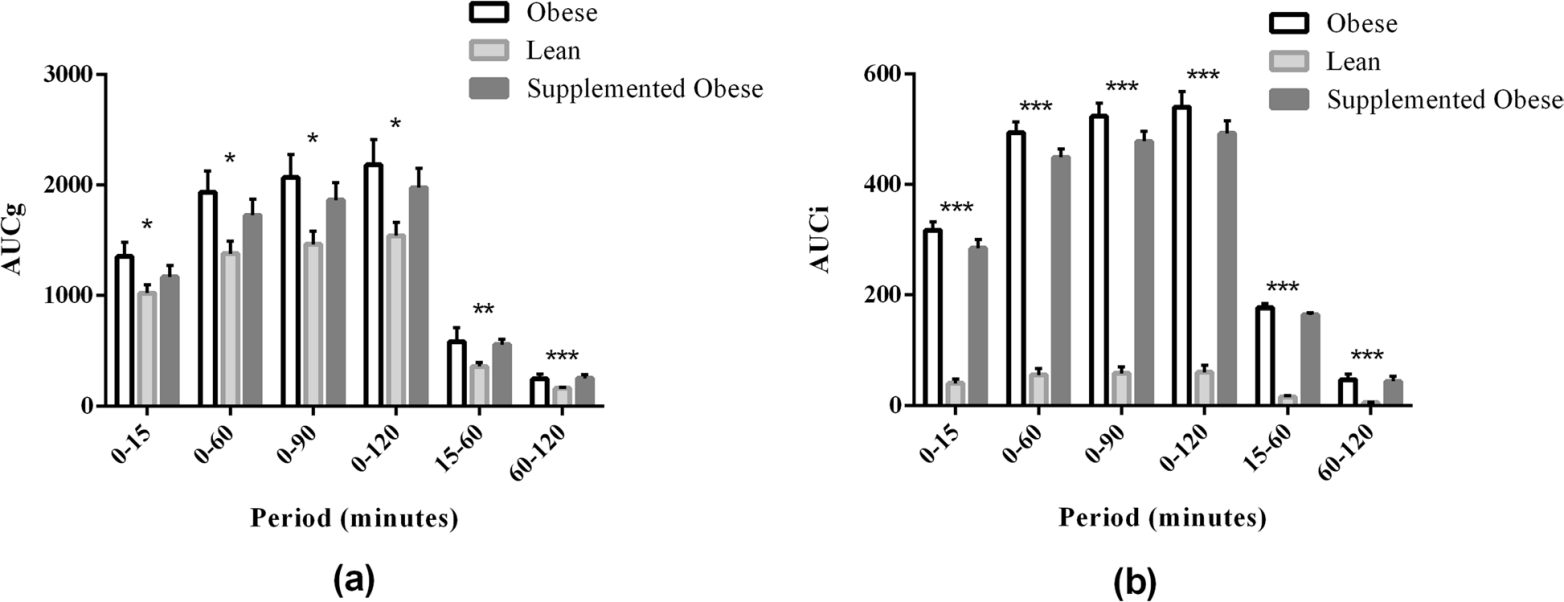
Area under the plasma glucose curve (AUCg) (**a**) and serum insulin curve (AUCi) (**b**) obtained during the intravenous glucose tolerance test of the experimental groups

Regarding the area under the glucose curve (AUCg), results for OG and SOG were not different (*p *> 0.05) for all periods (Fig. 2). However, OG was different from LG and, after consumption of the test diet, the SOG was not different form the LG for almost all of the periods with exception for 60-120 minutes (*p *= 0.032) and 15-60 minutes (*p *= 0.007). Serum insulin concentrations were increased in groups OG and SOG than in LG at all intervals (*p *< 0.05). Figure 2 also shows the values of the area under the insulin curve (AUCi).

**Fig. 3:**
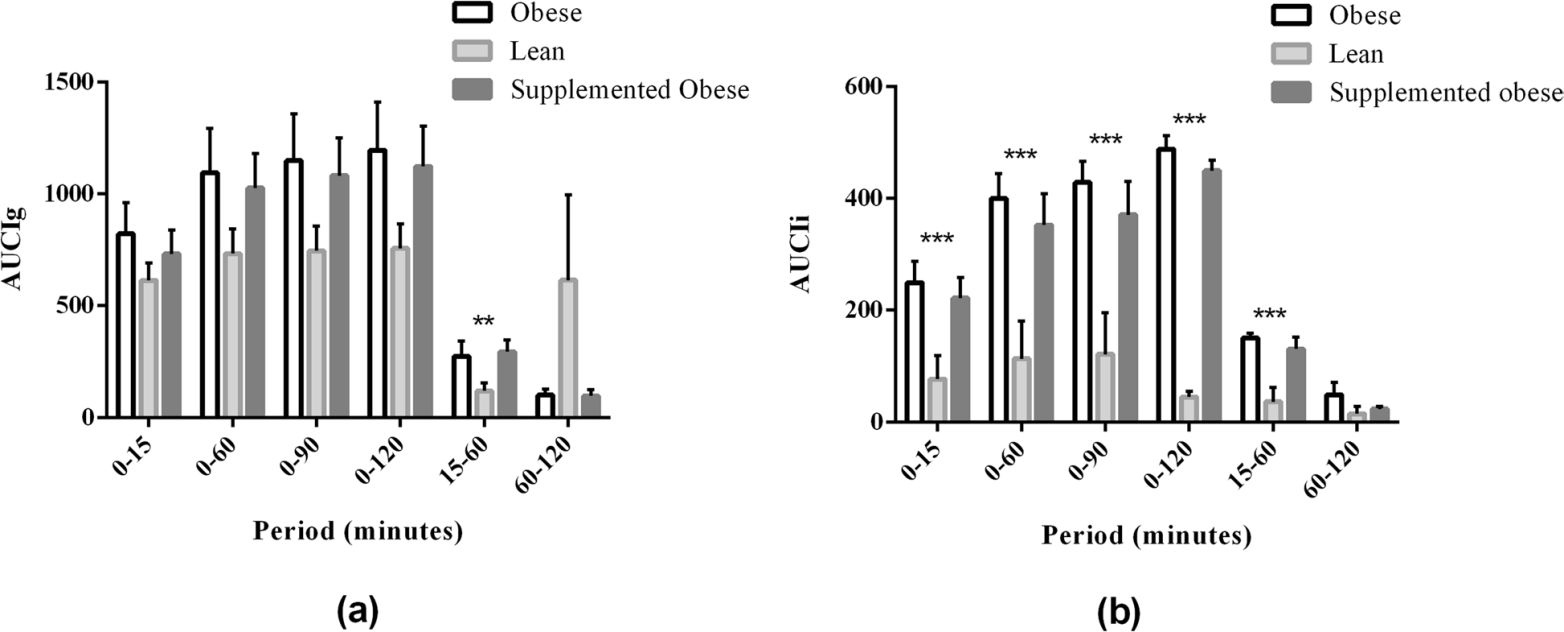
Area under the plasma glucose increment (AUCIg) (**a**) and serum insulin increment (AUCIi) (**b**) curves obtained during the intravenous glucose tolerance test of the experimental groups

Area under the plasma glucose increment curve (AUCIg) was not different among the groups (*p *> 0.05), except for at 15-60 minutes (*p *= 0.021) in LG and SOG (Fig. 3). Regarding the area under the insulin increment curves (AUCIi), the only time point that did not show any variation in insulin secretion was the 60-120 minutes interval; in contrast, all other AUCIs were increased in OG and SOG than in LG (*p *< 0.05).

As described in Table 1, basal and mean blood glucose values were higer in OG than in LG and SOG (*p *< 0.05). Similar results were obtained for basal insulin levels, although SOG had intermediate values (*p *< 0.05).

**Table 1:** Values (means ± standard error) of basal glucose, basal insulin, minimum glucose, maximum glucose, mean blood glucose and median values (minimum; maximum) of K, T½, and ΔI/ΔG of the experimental groups

Variables	OG	Experimental groups	SOG
		LG	
Basal glucose (mg/dL)	106.29 ± 5.74^A^	81.23 ± 3.71^B^	87.14 ± 3.83^B^
Basal insulin (mUI/mL)	25.85 ± 1.23^A^	7.85 ± 0.42^C^	21.70 ± 0.67^B^
Minimum glucose (mg/dL)	84.00 ± 8.88^A^	71.57 ± 2.32^A^	77.57 ± 5.48^A^
Maximum glucose (mg/dL)	335.43 ± 34.53^A^	299.29 ± 22.18^A^	282.14 ± 22.30^A^
Mean blood glucose (mg/dL)	207.81 ± 21.49^A^	146.92 ± 10.92^B^	188.00 ± 16.54^B^
K^1^ (%)	81.79 (72.40; 89.90)^a^	11.67 (4.20; 22.30)^c^	75.10 (68.00; 79.50)^b^
T½ (minutes)^2^	15.00 (5.00; 15.00)^a^	2.50 (2.50; 7.50)^b^	15.00 (2.50; 15.00)^a^
ΔI/ΔG^3^	0.38 (0.20; 0.49)^a^	0.04 (0.02; 0.08)^b^	0.35 (0.25; 0.71)^a^

Reference ranges: Glucose = 65-118mg/dL; Insulin = 5-20mUI/mL [28]. ^1^K. percentage of glucose disappearance; ^2^T½. time for glucose to reduce to half; ^3^ΔI/ΔG. insulinogenic index; A, B, C – Averages followed by the same uppercase letters in the rows do not differ, as determined by Student’s t-test (p < 0.05); a, b, c – Medians followed by the same lowercase letters in the rows do not differ, as determined by the Wilcoxon test (p < 0.05). OG: obese group, LG: lean group, SOG: supplemented obese group.

The results for percentage of glucose disappearance (K), glucose half-life (T½), insulinogenic index (ΔI/ΔG) were not normally distributed and were evaluated by Wilcoxon test (Table 1). The rate of glucose removal, as evaluated by K was different (*p *< 0.05) among the three groups, and the T½ and ΔI/ΔG (*p *< 0.05) of LG were different in the other groups.

Cholesterol and triglyceride serum concentrations were higher in the OG than compared to LG, and beta-glucan intake affected these variables (Table 2).

**Table 2:** Serum concentration (mean ± standard error) of amylin, glucagon, leptin, inflammatory adipocytokines and appetite-regulating hormones in the experimental groups

Variables	OG	Experimental groups	SOG
		LG	
Cholesterol (mg/dL)	286.28 ± 26.06^A^	154.0 ± 14.66^B^	191.57 ± 24.74^B^
Triglycerides (mg/dL)	151.00 ± 12.28^A^	86.28 ± 8.70^B^	108.85 ± 9.32 ^B^

Reference ranges: Cholesterol = 135-270mg/dL; Triglycerides = 20-112mg/d L[28]. A, B - Averages followed by the same letter in the rows do not differ from each other, as determined by Student’s t-test (p<0.05). OG: obese group, LG: lean group, SOG: supplemented obese group.

No differences in serum amylin and glucagon concentrations were observed for any of the experimental groups (Table 3). Leptin values were higher in the OG and SOG than in the LG (*p *< 0.05). Similar results were also obtained for serum C-reactive protein concentrations (Table 3). Beta-glucan supplementation resulted in similar serum TNF-α concentrations in LG and SOG compared to OG (*p *< 0.05). Beta-glucan also affected the appetite-regulating hormone GLP-1 (Table 3) and it was lower in LG and OG than in SOG (*p *< 0.05).

**Table 3:** Serum concentration (mean ± standard error) of amylin, glucagon, leptin, inflammatory adipocytokines and appetite-regulating hormones in the experimental groups

Variables	OG	Experimental groups	SOG
		LG	
Amylin (pg/mL)	3.05 ± 1.18^A^	3.98 ± 1.80^A^	5.63 ± 3.52^A^
Glucagon (pg/mL)	116.75 ± 50.55^A^	154.54 ± 37.44^A^	105.21 ± 51.78 ^A^
Leptin (pg/mL)	10000.14 ± 2476.03^A^	939.86 ± 375.25^B^	16714.00 ± 4098.69^A^
IL-6 (pg/mL)	31.9 ± 23.46^A^	5.5 ± 2.87^B^	11.3 ± 6.41^A^
C-reactive protein (pg/mL)	8.8 ± 3.88^A^	3.1 ± 2.23^B^	6.1 ± 2.98^A^
TNF-α (pg/mL)	4.09 ± 1.18^A^	0.7 ± 0.35^B^	0.9 ± 0.49^B^
PYY (pg/mL)	75.30 ± 44.32^A^	86.46 ± 48.00^A^	179.78 ± 46.18^A^
GLP-1 (pg/mL)	4.32 ± 3.04^B^	1.01 ± 0.38^B^	14.53 ± 6.50^A^

Reference range for C-reactive protein: up to 10pg/mL [29]. A, B - Averages followed by the same letter in the rows do not differ from each other, as determined by Student’s t-test (p<0.05). OG: obese group, LG: lean group, SOG: supplemented obese group.

## Discussion

The effects of beta-glucans on glycemic control are poorly understood in dogs and few studies have evaluated how the addition of this polysaccharide affects the physiology of obese dogs.

In healthy humans, beta-glucan consumption improved glycemic control and/or insulin response [30, 31], and this has also being noticed in obese human patients and those with type II diabetes mellitus [32, 33]. Other studies have failed to identify this improved insulin response following beta-glucan supplementation in animal models [34], in people [35] and in dogs [22]. Notably, most studies used high doses of beta-glucans with vegetal origin.

Ferreira et al. [22] evaluated the effects of plant-based beta-glucan supplementation on fasting plasma glucose concentrations, 60 and 120 minutes after food consumption, and also observed no changes in this parameter after 28 days, corroborating Vetvicka and Oliveira’s study with healthy animals [23]. However, the authors of this study believe that the hypoglycemic compensatory effect appears to occur in non-physiological situations as in the glucose tolerance test performed in this study in animals with previous alteration of glycemic homeostasis, such as insulin resistance in obese animals. In healthy animals whose glycemic control is adequate, beta-glucan does not appear to produce significant effects.

In obese dogs, the diet supplemented with 0.1% beta-glucan induced important changes in several glycemic variables, basal serum insulin levels, and triglyceride levels, and these parameters resembled those of the LG. The beta-glucan supplementation also reduced basal insulin concentration in the SOG, although these values remained higher than those of LG.

K and T½ were both altered in this study. These results indicate that the experimental groups were composed by animals with impaired glucose tolerance according to Kaneko [28]. The lower glucose tolerance found in obese dogs was expected since the selection of animals has prioritized those with insulin resistance, although importance must be given to the fact that glucose intolerance was indeed verified in the obese animal group and that beta-glucan was able to significantly reduce this parameter.

Similar as in the present study, a mouse study evaluated the effect of modest doses of yeast-derived beta-glucans, and the results demonstrated that no direct glycemia effects exist in normal homeostatic states. However, animals with experimentally induced hyperglycemia had significantly reduced glycemia [23]. Only two studies were found that evaluated the effects of beta-glucans on blood glucose in dogs. Vetvicka and Oliveira [23] evaluated the use of two different sources of beta-glucans and observed that in healthy animals, after 14 days of dietary supplementation, beta-glucan did not induced significant glycemic effects. However, when animals with experimentally induced hyperglycemia were evaluated, beta-glucans reduced glycemic values to the reference range of healthy dogs after seven days of feeding.

Some studies have attempted to elucidate the triglyceride-lowering properties of soluble fibers, including beta-glucans. These ingredients may possibly delay triglyceride absorption in the small intestine [36] and reduce rate of glucose absorption [37], and hypertriglyceridemia is induced via glucose by de novo lipogenesis process. In addition, the direct inhibition of lipogenesis by soluble fibers is suggested as an explanatory mechanism [38]. An in vitro study demonstrated that beta-glucans from barley and oatmeal inhibited long-chain fatty acid and cholesterol absorption and, in rats, repressed several genes involved in lipogenesis and lipid transport [39].

As one of the main purposes of this study was to evaluate the application of this fiber for obese dogs in order to benefit satiety during weight loss, alterations related to hormones and adipokines evolved in this control are of great interest as a sizeable portion of dogs are overweight and obese worldwide [4, 40] and, simultaneously, many of those do not respond adequately to weight loss or regain the weight after treatment [41].

Amylin is an insulin co-secreted hormone that has glucagon suppressive activity and reduces gastric emptying, which could interfere in satiety modulation. In our results, no correlation was found between amylin concentrations and obesity or between amylin concentrations and the effects of beta-glucan supplementation. No studies had evaluated amylin concentrations in obese dogs, making further discussion of our results difficult. PYY is a great appetite inhibitor and satiety hormone, and this peptide belongs to a group of peripheral hormones responsible for regulating food intake through hypothalamic and cerebral signaling [42], displaying an important role in obesity control [43].

Regarding the ability of beta-glucans to increase the release of PYY and, despite the large numerical difference between the mean PYY value in OG compared to SOG and LG, no difference was observed in the present study, as well as in the study published by Ferreira et al. [22].

In another study with dogs, similarly to our results, the administration of a diet composed of high soluble fiber levels was not sufficient to increase the serum PYY concentrations in obese animals [44, 45].

Both PYY and GLP-1 increase the gastric emptying time and small intestine transit time [45]. These effects may lead to prolonged gastric distension and, consequently, signs of satiety [44, 45], as well as delayed nutrient contact with the small intestinal receptors involved in maintaining satiety. Prolonged gastric emptying can also impair starch digestion and consequently glucose absorption, hence improving post-prandial glucose and insulin concentration stability [46, 48].

GLP-1 is an anorectic metabolite and glycemic control regulator that exerts cytoprotective, antioxidative and hemodynamic actions, inhibits gastric emptying and is believed to be responsible for reducing hunger and increasing satiety [47, 48].

In the light of the current knowledge regarding prebiotic properties of beta-glucan and obesity-related dysbiosis that increase the energy harvest, the overall results obtained in this study may also be related to the modulation of gut microbiota and, as a consequence, the modulation of their fermentation products (short chain fatty acids) as well [49–51].

Short-chain fatty acids produced by fiber fermentation affect satiety primarily by releasing appetite-regulating hormones, such as the aforementioned PYY, GLP-1 but also ghrelin. However, other unknown mechanisms, independent of short-chain fatty acids, may be involved in regulating intestinal incretins through beta-glucan consumption. Since research in this area is still limited, these mechanisms require further investigation.

Based on several previous human studies, substantial evidence exists that suggests a minimal beta-glucan level is required (averaging 4 to 6 g) to perform appetite-regulating effects through circulating incretins [52–54]. However, pioneering studies that have evaluated the direct effect of beta-glucans on satiety parameters in dogs and that have shown positive results with low inclusion content (0.1% in the formulated diet) are quite scarce.

These findings, when correlated with the practical observations in this study, explain why four out of the seven obese dogs presented with leftover food as the study progressed and why none of the dogs manifested begging behavior, attempting to obtain more food.

Finally, concerning to the physiological consequences of obesity, since fat tissue is responsible for the production of many inflammatory mediators and adipocytokines [55], controlling the effects of low grade inflammation as well as the perception of energy flow within the cell during the weight loss program is of great benefit for the obese patient in many ways.

Of the known adipocytokines, the most studied is leptin. Although leptin is secreted by adipocytes, increased secretion is based on the energy flow within adipocytes and circulating leptin concentrations are correlated with fat mass [56]. Thus, as reported in the literature, OG had increased serum leptin concentrations.

In companion animals, other inflammatory obesity markers and their relationship with weight loss have not been well established. However, adipose tissue is known to synthesize and release other pro-inflammatory factors, such as TNF-α, IL-6, IL-1, C-reactive protein and monocyte-1 chemotactic protein (MCP-1) [57, 58]. Alteration in the concentrations of these adipokines are directly or indirectly associated with physiological, metabolic and immunological changes, which results in the increased production and circulation of inflammation-related factors. For example, TNF-α, a cytokine that is known to promote cachexia and is produced by macrophages and adipocytes, and IL-6, which has marked catabolic function, are also involved in insulin resistance in type II diabetic patients [59]. Some authors have demonstrated that these changes and the secretion profile of these cytokines tend to be rectified with weight loss [12, 60, 61].

Bastien et al. [61] observed that, throughout weight loss programs, pro-inflammatory cytokine concentrations decreased, which are related to the low-grade inflammation induced by obesity and associated comorbidities. Vitger et al. [60] evaluated immunometabolic parameters in overweight dogs during weight loss by evaluating the levels of circulating leptin, C-reactive protein, and other adipokines. The authors concluded that weight loss improved health indicators.

The results of our study corroborate those in the literature, as inflammatory adipokine levels were lower in lean animals than in obese animals. However, the SOG (even without performing a weight loss program) had intermediate values of IL-6 and C- reactive protein. Serum TNF-α concentrations were lower in SOG than in the OG and were similar to those of the LG.

In relation to some evaluated variables, reference values ​​are not well established for dogs in veterinary literature, so that justifies the creation of a lean group in order obtain those results in lean dogs living under similar conditions in order to make better comparisons. Durocher et al. (2008) [62] found values between 28-55pg / mL for glucagon in healthy dogs and 19-414pg / mL for diabetic dogs. Park et al. (2014) [63] found that mean serum concentrations of leptin in lean and obese dogs were, respectively, 2640 ± 2640pg / mL and 10.290 ± 1870pg / mL. Regarding serum concentrations IL-6 and TNF-α, Kim et al. [64] evaluating dogs with myxomatous mitral valve disease, did not observe concentrations of these variables in healthy dogs (their control group). As for the remaining, no reference values are available.

## Conclusions

Inclusion of 0.1% beta-glucan in the diet reduced basal and mean plasma glucose concentration, basal insulin, triglyceride and cholesterol concentrations in obese dogs to values similar to those of lean dogs. For the other glycemic variables evaluated, intermediate values were found between supplemented obese dogs and the remaining groups; In addition, supplementation increased GLP-1 concentration and reduced circulating concentrations of TNF- α.

## Methods

### Animals, diets and experimental design

Dogs were selected at a private veterinary clinic located in Pirassununga (São Paulo, Brazil). The study was conducted at the fall-winter season, the weather is mainly dry and temperature ranged from 18 to 22°C. The animals had been previously evaluated with a complete physical examination and hematological exams, including assessments of complete blood count and liver and renal function. Dogs that presented with any laboratory abnormalities or clinical signs indicative of endocrine disease were evaluated through hormonal exams (free thyroxine by equilibrium dialysis, thyroid stimulating hormone and basal cortisol) and were excluded from the experimental groups if they were positive for endocrine disease.

The evaluation of insulin sensitivity was calculated by the HOMA (homeostasis model assessment) index, which considers the paired basal insulin with basal glucose levels, according to the equation: HOMA score = {basal serum insulin (mU/L) × {basal plasma glucose (mg/dL)}/40 5 [27] and animals are considered insulin resistant if HOMA values are higher than 2.4.

Three experimental groups were created to carry out the study. OG (*n *= 7) was composed by male (*n* = 1) and female (*n* = 6) dogs aged 4 to 10 years old (7.14 ± 0.85) with body condition score (BCS) of 8 or 9/9, according to the scale described by Laflamme [26] and all of which had insulin resistance. Dog breeds were golden retriever (2), Labrador retriever (2), poodle (1) and mixed breed (2). The dogs included in the OG had values higher than 2.4 and were classified as insulin resistant.

The LG (*n* = 7) group was composed by male (*n* = 3) and female (*n* = 4) dog aged 1 to 4 years old (2.4 ± 0.53 years old) with ideal body condition (BCS = 5) in order to provide a reference for normality of the parameter evaluated in owned dogs. Dog breeds were border collie (2), brazilian terrier (2), mixed breed (2), shih tzu (1).

Finally, the third group, also known as SOG, were the same dogs from OG, although after 90 days of consumption of diet supplemented with 0.1% beta-glucans^1^. The dose used was proposed by the manufacturer after internal trials.

**Fig. 4:**
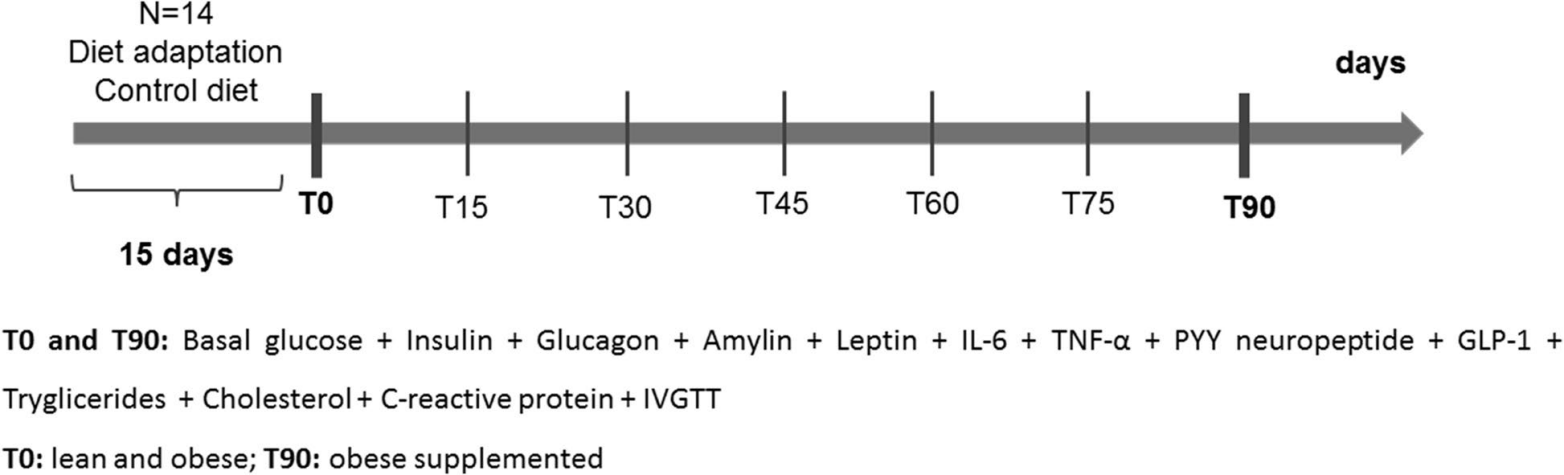
Timeline of the experimental procedures. T0=beginning of consumption of experimental diet by OG; T90=last day of consumption of the experimental diet

To standardize the diet since all dogs lived in different households, all animals in OG and LG were fed during a 15-day period with an experimental maintenance diet (control diet). After the diet acclimation period, collections and procedures for T0 started. OG received the experimental diet with beta-glucan supplementation (test diet) for 90 days, and then, consequently, composed SOG. The experimental diet compositions are described in Table 4 and were developed following recommendations from the Association of American Feed Control Officials [65] for adult dogs and the timeline of the experiment is illustrated in Figure 4.

**Table 4:** Experimental diet composition

Nutrient (%)	Control diet	Test diet
Moisture	7.8	7.8
Crude protein	21.73	22.42
Fat	7.44	7.61
Crude fiber	4.65	5.14
Ash	10.3	9.2
Nitrogen-free extract	48.09	47.83
Beta-glucans^2^	0	0.1

Ingredients: ground whole grain corn, brewers rice, ground whole grain sorghum, chicken by-product meal, rice bran, beet pulp, meat and bone meal, soybean meal, chicken fat, fish oil, hydrolyzed liver flavor, salt, premix^B^, antifungal^C^, antioxidant^D^; ^2^Product^A^ composed by 70% of yeast beta-glucan.

All animals received sufficient amounts of diet to meet the daily energy requirements (DER) [29] according to the following equation: DER = 95 x (body weight)^0.75^.

Since the intention was not to promote weight gain or weight loss during the experimental period, animals were weighed every 15 days for monitoring and adjustments in caloric intake if necessary.

### Biochemical and hormonal analysis

Analyses were performed at the beginning of the study, after the diet standardization (T0) in OG and LG, and at the end of the study (T90), after SOG had finished the test diet. Animals were fasted and cannulated via the cephalic vein with an intravenous peripheral catheter after a 12-hour fasting. Subsequently, 0.5mL blood samples were taken to evaluate glycemia; 2.0mL samples were collected to determine the serum concentrations of insulin, amylin, glucagon, leptin, IL-6, C-reactive protein, tumor necrosis factor alpha (TNF-α), polypeptide Y (PYY), and GLP-1; and 2.5mL samples were used to assess serum cholesterol and triglyceride concentrations, in addition to evaluating hepatic and renal function.

Plasma glucose concentrations were assessed by glucose oxidase assays (GOD-ANA, Labtest Diagnostica S.A., Lagoa Santa, Brazil), and serum insulin concentrations were analyzed by radioimmunoassay.

Levels of cytokine IL-6, C-reactive protein, and TNF-α were measured by a validated Milliplex® MAP cytokine panel for dogs (CCYTO-90K, Millipore, Massachusetts, USA).

Triglyceride, cholesterol, ALT and creatinine levels were determined using commercial kits (Labtest Diagnostica SA, Lagoa Santa, Brazil), and leptin, amylin, glucagon, Y polypeptide and GLP-1 levels were analyzed by Milliplex panels (CGTMAG-98K-03, Millipore, Massachusetts, USA).

### Intravenous glucose tolerance test (IVGTT)

Soon after the baseline blood collection, IVGTTs were performed, based on the method described by Mattheuws et al. [66]. A dose of 500mg of glucose/kg of body weight was infused to assess glucose tolerance and insulin sensitivity. To assess glycemia, aliquots of blood (0.5mL) were collected in tubes containing fluoridated EDTA, and 2.0mL aliquots were used to determine serum insulin concentrations at 0, 2.5, 5.0, 7.5, 10, 15, 30, 45, 60, 90 and 120 minutes after glucose infusion.

### Calculations and statistical analysis

Analyzed data are expressed as mean ± SEM. The calculation procedures used for interpreting IVGTT data were described in the literature by Mattheuws et al. [66]. The data obtained during the test were analyzed as described by De-Oliveira et al. [67] and Brunetto et al. [9]. The area under the curve (AUC) was calculated in Prism software (GraphPad Prism, version 5) by numerical integrations using the trapezoidal method with mean values at each time for all animals.

For statistical analysis, comparisons between groups were previously established and performed by Student's t-test for variables that met data normality assumptions. Variables that did not meet this assumption were analyzed by Wilcoxon nonparametric tests. Blood glucose concentrations, glucose increments, serum insulin concentrations and insulin increments were analyzed by means of analysis of variance of repeated measures for each time point. Multiple comparisons were assessed by Tukey’s test, and p<0.05 values were considered significant. The results were obtained using the SAS program.

## Data Availability

All data generated or analysed during this study are included in this published article.

## Footnotes

1 ^A^MacroGard, Biorigin, Lençois Paulistas, Brazil; ^B^Pettymeal, MCassab, São Paulo, Brazil; ^C^Amo Curb, Kemin, São Paulo, Brazil; ^D^Endox-5x, Kemin, São Paulo, Brazil.

## Abbreviations

AUCg: area under the curve of glucose
AUCi: area under curve of insulin
GLP-1: Glucagon-like peptide-1
HOMA: homeostasis model assessment
AUCIg: area under glucose increment curve
AUCI: area under insulin increment curve
IL-1: interleukin 1
IL-6: interleukin 6
IVGTT: Intravenous glucose tolerance test
K: percentage of glucose disappearance
LG: lean group
OG: obese group
PYY: polypeptide Y
SOG: supplemented obese group
T½: glucose half-life
TNF-α: tumor necrosis factor alpha
ΔI/ΔG: insulinogenic index
